# Interactions between β-Lactoglobulin and 3,3′-Diindolylmethane in Model System

**DOI:** 10.3390/molecules24112151

**Published:** 2019-06-07

**Authors:** Cuina Wang, Xinhui Zhou, Hao Wang, Xiaomeng Sun, Mingruo Guo

**Affiliations:** 1 Key Laboratory of Dairy Science, Northeast Agricultural University, Harbin 150030, Heilongjiang, China; wangcuina@jlu.edu.cn (C.W.); zhouxh15@mails.jlu.edu.cn (X.Z.); jlu_wh@126.com (H.W.); sunxm15@mails.jlu.edu.cn (X.S.); 2 Department of Food Science, College of Food Science and Engineering, Jilin University, Changchun 130062, Jilin, China; 3 Department of Nutrition and Food Sciences, College of Agriculture and Life Sciences, University of Vermont, Burlington, VT 05405, USA

**Keywords:** 3,3′-diindolylmethane, β-lactoglobulin, spectroscopic analysis, molecular docking

## Abstract

The compound 3,3′-diindolylmethane (DIM) has a broad spectrum of anticancer activities. However, low stability and bioavailability limit its application. Elucidating interactions between DIM and β-lactoglobulin (β-LG) may be useful for fabricating whey protein-based protecting systems. Interaction with DIM increased the diameter and absolute zeta potential value of β-LG. UV-absorption spectra suggested that there was a complex of DIM and β-LG. β-LG showed enhanced fluorescence intensity by complexing with DIM with a binding constant of 6.7 × 10^5^ M^−1^. Upon interaction with DIM, β-LG was decreased in secondary structure content of helix and turn while increased in β-sheet and unordered. FT-IR spectra and molecular docking results indicated the roles of hydrophobic interaction and hydrogen bond for the formation of DIM and β-LG nanocomplexes. Data suggested that β-LG may be a good vehicle for making a protein-based DIM protection and delivery system due to the tight binding of DIM to β-LG.

## 1. Introduction

The bioactive compound 3,3′-diindolylmethane (DIM) is present in cruciferous vegetables. It is a member of indoles with two imido groups and two benzene rings. DIM can bind to Aryl hydrocarbon receptor and induce expression of gene *CYP1A1* [1], which can significantly inhibit breast cancer cell proliferation [2]. Due to its bioactivity, DIM is becoming more and more popular in functional foods and the pharmaceutical industry in recent years. However, its low stability and poor oral bioavailability is a major challenge for its broad application. Direct incorporation of DIM into food or intraperitoneal injection will result in its functionality loss [3] and high lipophilicity may be responsible for its poor oral bioavailability [4]. Previous study has shown that zein/carboxymethyl chitosan nanoparticles can be used to improve the sensitivity and release properties of DIM [5].

β-Lactoglobulin (β-LG) is a main globular whey protein with a proportion of 50–60% [6]. β-LG is a small water-soluble protein [7] with molecular weight of about 18.40 kDa and total amino acid residues of 162 [8]. This protein has a barrel structured by eight antiparallel β-strands and an α-helix located at the outer surface of the barrel [9]. It is classified in the lipocalin-protein family, which has a strong affinity towards various ligands [10]. It was found to be able to bind retinol as early as 1976 [11]. Recent research showed that it can bind and deliver linoleate [12], lutein [13], curcumin [14], fucoxanthin [15], astaxanthin [16], and β-carotene [17].

Protein-binding mechanisms for hydrophobic molecules vary from hydrophobic interaction and van der Waals attraction to hydrogen bond [15]. For β-LG, hydrophobic interaction may be the main mechanism. It has three hydrophobic binding sites. One is in the cavity of the barrel. Under certain conditions, it adopts a conformation which allows the ligands to enter into the barrel [18]. The other two are located on the surface cleft hydrophobic site between the α-helix and the barrel formed by β-strands [19]. The specific binding site on β-LG is determined by the structure of the binder. Fatty acids with linear structures bind on the cavity of the barrel [18], retinol and retinol acid with ring and linear structure bind on both the surface cleft and cavity [20], and polar aromatic compound ellipticine binds on the surface site [21].

β-LG is a good encapsulating system for nutraceuticals [22]. Whey protein as a carrier can improve the bioactivity of hydrophobic ligands by improving the stability of ligands [23] or in vivo transfer efficiency [16,24]. In addition, β-LG is a well-known food allergen and studies have shown that binding of β-LG to a number of compounds could play a coadjutant role in the onset of immune responses and reducing allergy [25,26]. Based on the structures of DIM and β-LG, fabricating whey protein-based DIM nanoparticles may be a good way to improve the stability and bioavailability of DIM. This study aims to investigate interactions between DIM and β-LG by measuring diameter, zeta potential, UV-vis absorbance and fluorescence spectra, and Fourier transform infrared (FT-IR) and far-ultraviolet circular dichroism (Far-UV CD) spectra. The binding process was also studied using molecular docking.

## 2. Results and Discussion

### 2.1. Effects of DIM on Particle Size of β-LG in Solution

Effects of DIM on the PDI (polydispersity index) and *D*_h_ of β-LG were studied and the results are shown in Table 1. Samples had PDI values ranging from 0.27 to 0.34, indicating monodispersed systems. Addition of DIM broadened the particle size distribution significantly (*p* < 0.05). However, no significant difference was found between samples with different levels of DIM. As shown in Table 1, free β-LG had a Z-average hydro-diameter of about 4.86 nm. It is reported that β-LG monomer has a diameter of about 2 nm and aggregates into a dimer in solution at medium pH and room temperature [27]. Low concentration of DIM (50 μM) did not affect the particle size of β-LG significantly (*p* > 0.05). However, the diameter was significantly increased (*p* < 0.05) when DIM was increased to 100–200 μM, indicating that DIM may absorb on or enter into the β-LG molecules, giving an increase in hydrodynamic diameter. β-LG has three binding sites which can bind hydrophobic compounds [10]. At alkaline condition, entrance to the cavity of barrel was open [28] and ligands can enter the cavity as well as absorb on the surface. Therefore, high levels of DIM may enter or absorb into the hydrophobic core through hydrophobic and hydrogen bonds while low levels of DIM may not be enough to generate detected significant changes in diameter.

### 2.2. Effects of DIM on Zeta Potential of β-LG

Effects of DIM on the zeta potential of β-LG were determined and the results are shown in Table 1. Free β-LG showed a negative zeta potential value (−8.63 ± 0.50 mV), indicating negative surface charges carried samples. The results were reasonable since the isoelectric point of β-LG is about 5.3 [29]. Addition of DIM (50 μM) decreased the zeta potential of β-LG to −17.93 mV, indicating that DIM may be attached on the molecules. However, further increase in DIM concentration from 100 to 200 μM did not affect the zeta potential significantly (*p* > 0.05), suggesting that the excessive DIM may be entrapped in the core, which did not affect the surface charge of β-LG.

### 2.3. Effects of DIM on UV-Absorption Spectra of β-LG

**Figure 1:**
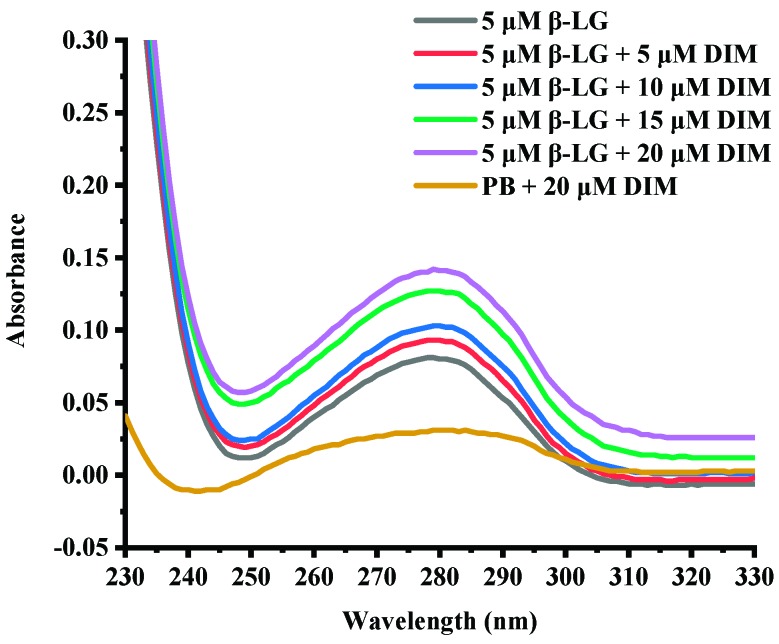
UV-absorption spectra of β-LG solution in presence of DIM (0–20 μM).

UV-absorption spectra are commonly used to provide information about protein structure changes due to interaction with another compound [30]. Figure 1 shows the UV-absorption spectra of β-LG in buffer solution in the presence of various levels of DIM. Free β-LG showed a peak at about 280 nm due to the conjugate double bonds in tyrosine (Tyr) and tryptophan (Trp) residues, which was typical for proteins containing aromatic amino acids [30]. Complexation with DIM did not change the maximum absorbance wavelength of β-LG. However, the absorbance was increased with DIM concentration increasing from 5 to 20 μM. The absorbance at near 280 nm depends on Tyr and Trp content [31]. The results suggested that DIM did not significantly change the polarity around protein.

### 2.4. Effects of DIM on Fluorescence Emission Spectra of β-LG

**Figure 2:**
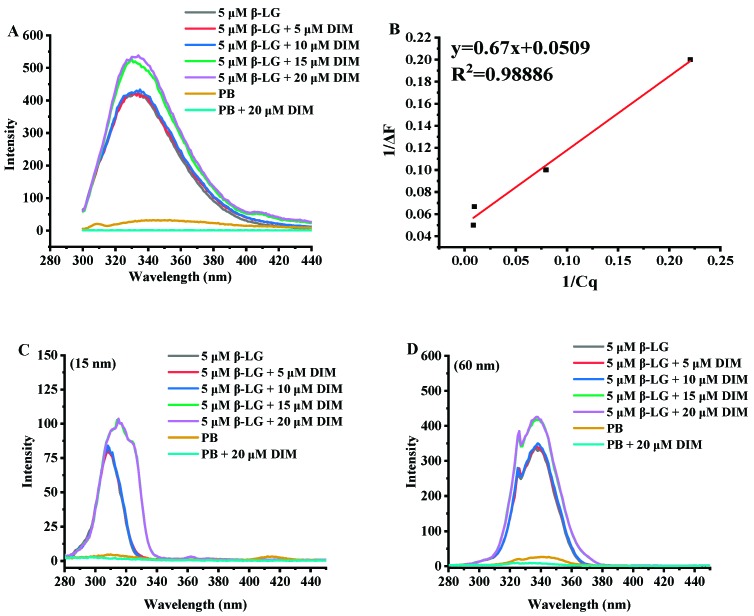
Fluorescence emission spectra (**A**) and synchronous fluorescence emission spectra (15 nm, **C**; 60 nm, **D**) of β-LG with 0–20 μM DIM. (**B**) Linear plot of 1/Δ*F* vs. 1/*C_q_* according to Equation (1).

Fluorescence analysis can provide useful information about interactions between proteins and small molecules due to the sensitivity of intrinsic fluorescence to the microenvironment changes around proteins [32]. Thus, all samples were assessed for fluorescence spectra and the results are shown in Figure 2.

Figure 2A shows the fluorescence emission spectra of β-LG and β-LG with various levels of DIM. β-LG had a fluorescence emission maximum (λ_max_) at 330 nm at excitation wavelength of 280 nm. Similar results were reported by a previous study [33]. After addition of DIM, the wavelength of maximum fluorescence intensity shifted towards a larger wavelength. Fluorescence red-shifts indicated that more Trp residues may be exposed to the solvent [34].

β-LG is able to bind various hydrophobic or amphiphilic compounds such as polyphenol and fatty acids [12,35]. Most of the compounds were observed to be able to quench the fluorescence intensity of β-LG in solution. However, DIM was observed to enhance the intrinsic fluorescence intensity of β-LG, suggesting changes in the exposed content of Trp to solvent. Based on the fluorescence enhancing effect equation (Figure 2B, R^2^ = 0.98886), the binding constant was calculated to be 6.7 × 10^5^ M^−1^. The binding constant is much higher than those reported in previous studies based on fluorometric experiments. Studies on interaction of tea polyphenols and β-LG showed that polyphenols bound β-LG via both hydrophilic and hydrophobic interactions with binding constants ranging from 2.2 × 10^3^ to 1.3 × 10^4^ M^−1^ [35]. The binding constant for curcumin is 1.3 × 10^5^ M^−1^ [10] and for fucoxanthin is 2.6 × 10^4^ M^−1^ [15].

β-LG monomer is a single polypeptide with 2 Trp residues (19 and 61) and 4 Tyr residues (20, 42, 99 and 101) which possess intrinsic fluorescence. Synchronous fluorescence was also conducted to study the effect of DIM on the microenvironment of β-LG by separating the emission peaks of Tyr and Trp residues. At a wavelength interval of 15 nm, only Tyr residues show fluorescence emission, and at a wavelength interval of 60 nm for Trp [36]. As shown in Figure 2C,D, the synchronous fluorescence spectra at a wavelength interval of 60 nm showed a stronger intensity than those at interval of 15 nm, indicating that Trp was dominant in the total fluorescence emission spectra. The results were consistent with a previous study that showed Trp exhibited stronger intrinsic fluorescence than Tyr [33]. Trp 19 is inside the cavity of the barrel and Trp 61 is located on the surface of β-LG near the cavity of the barrel [37]. The reason for the stronger intensity may be that the hydrophobic interactions between DIM and β-LG more significantly influenced Trp 19.

### 2.5. Effects of DIM on Second Structure of β-LG

**Figure 3:**
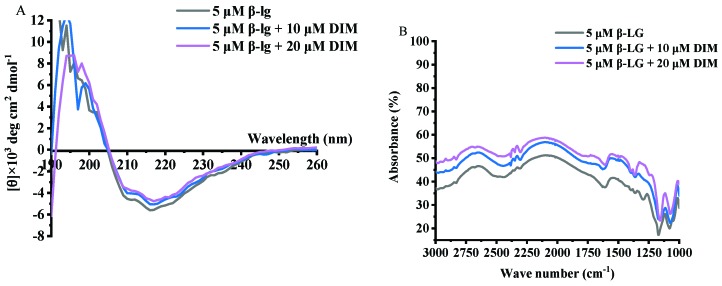
Far-UV CD (**A**) and FT-IR spectra (**B**) of β-LG with 0, 10 and 20 μM DIM.

Samples of β-LG and β-LG with 10 or 20 μM DIM were measured for CD spectra and the results are shown in Figure 3A and Table 2. Free β-LG contained 16.15% helix, 34.85% β-sheet, 22.55% turn and 26.50% unordered structure, indicating that β-sheet is the dominant secondary structure. The results were consistent with a previous study [38]. Upon interaction with DIM, the helix content decreased from 16.15% to 8.75% and 5.35%, and turn content decreased from 22.55% to 19.35% and 16.95%. Meanwhile, the β-sheet and unordered structure increased from 34.85% to 38.3% and 47.90% and from 26.50 to 33.40% and 29.75%, respectively. The increased β-sheet may be due to the complex of β-LG with DIM [32]. Similar results were reported for tea polyphenols [35].

### 2.6. Effects of DIM on Fourier Transform Infrared (FT-IR) Spectra of β-LG

DIM is a member of indoles with two imido groups and two benzene rings. Theoretically, hydrogen bonding and hydrophobic interactions between β-LG and DIM may be involved. To investigate the possible interactions between DIM and β-LG, FT-IR spectra for samples of β-LG and β-LG with 10 or 20 μM DIM were conducted and the results are shown in Figure 3B. β-LG showed characteristic absorption bands at 1548 cm^−1^, which is attributed to N–H plane bending and C-N stretching vibration (Amide II) [39]. Addition of DIM did not shift the wavelength but increased the intensity of the absorbance, indicating the introduction of the N–H group of the DIM. The absorption band at about 1650 cm^−1^ represents Amide I, which is the C=O stretching vibration. The increase in the intensity of absorption indicated the exposure of the peptides. Increase in the intensity of Amide I and Amide II indicated the binding of DIM to the β-LG. Similar results were obtained by Hasni et al. [24] who found the increase in the intensity of Amide I and Amide II but no major shifting after the binding of lipid to β-LG. Both Amide I and Amide II are sensitive to the change of secondary structure [40]. Changes in the intensity indicated the alteration of the secondary structure of β-LG caused by DIM. The results were consistent with those of CD spectra results. Hydrogen bonding and hydrophobic force may be the main forces attributed to the interaction of protein and small hydrophobic substance [41]. C=O, N–H, C–N are often involved in the hydrophilic interaction and form hydrogen bonds. The absorption band in the region of 2800 to 3000 cm^−1^ is attributed to –C–H antisymmetric stretching vibration and is also involved in hydrophobic interaction [24]. Changes in the intensity indicated the involvement of hydrophobic interaction in the β-LG and DIM complexes.

### 2.7. Effects of DIM on microstructure of β-LG particles

**Figure 4:**
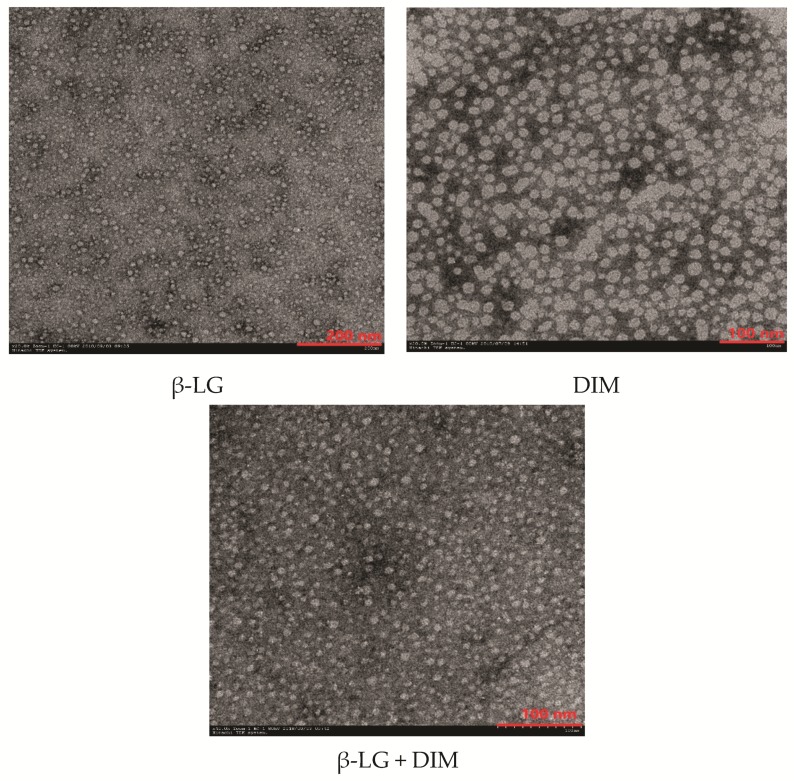
Transmission Electron Microscopy (TEM) micro photographs of β-LG, DIM and mixture of β-LG (5 μM) and DIM (20 μM).

To understand the effects of DIM on the microstructure of β-LG, morphological properties of β-LG, DIM and a mixture of β-LG (5 μM) and DIM (20 μM) were observed using Transmission Electron Microscopy (TEM) and the results are shown in Figure 4. Free β-LG has a spherical shape with a diameter of about 20 nm. The results are in consistent with those of a previous study [15]. DIM showed irregular shape at the scale of about 20 nm. Upon complexing, it seemed that the particles became more uniform and the edges of the particles blurred and the diameter increased compared to free protein. This suggested that DIM and β-LG interacted in the model system. The results were consistent with those determined by Dynamic Light Scattering (DLS) where particle size of β-LG was increased by interaction with DIM.

### 2.8. Molecular Docking between DIM and β-LG

**Figure 5:**
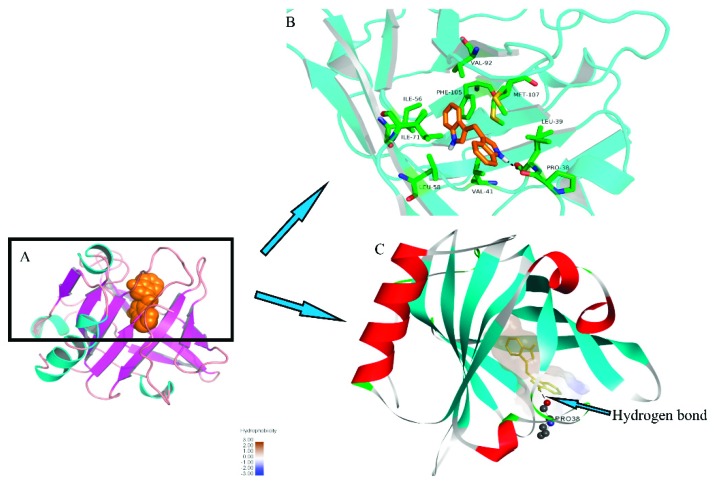
Molecular docking of DIM into β-LG. β-LG is shown colored in cyan (helix), magenta (sheet), and salmon (loop). The sphere model displays DIM colored orange. The stick models represent DIM and key amino acid residues, colored orange and green, respectively.

DIM was docked to the X-ray diffraction crystal structure of β-LG and the stereo view of docked structure were shown in Figure 5. As shown in Figure 5A, the DIM and β-LG complex was mainly stabilized via hydrophobic and hydrogen bonding, which was similar to those for FT-IR spectra. As shown in Figure 5B, DIM bound on the entrance of the calyx and Leu39, Val41, Ile56, Leu58, Ile71, Val92, Phe105, Met107 are responsible for the hydrophobic interactions. Docking results showed that only one aromatic amino acid (Phe 105) was involved in the hydrophobic interaction. This result may be connected with the results of UV-absorption spectra where no significant shift of maximum absorbance wavelength was observed for β-LG after addition of DIM. Figure 5C shows that there was a hydrogen bond between the –NH of DIM and carbonyl group of Pro38 residues in β-LG with an average binding distance of 2.226 Å and hydrogen bond angle of 133.034°. The estimated free energy of binding (ΔG_binding_) was −40.06 KJ/mol, which was much higher than that for fucoxanthin (−22.65 KJ/mol), indicating that DIM bound more tightly with β-LG than fucoxanthin [15]. The results are consistent with that for the binding constant.

## 3. Materials and Methods

### 3.1. Materials

β-Lactoglobulin (β-LG, ≥90% purity, lyophilized powder) was obtained from Sigma-Aldrich (St. Louis, MO, USA). The 3,3′-Diindolylmethane (>99% purity) compound was purchased from Luotian Pharmaceutical Co., Ltd. (Hubei, China). Potassium bromide was purchased from Sigma-Aldrich (St. Louis, MO, USA). All other chemicals were obtained from Beijing Chemical Works (Beijing, China).

### 3.2. Preparation of β-LG and DIM Solution

β-LG stock solution (5 μM) was prepared by dissolving β-LG powder slowly into phosphate buffer (PB) solution (pH = 7.0) and stirred under room temperature for 1 h for complete hydration. Stock solution of DIM (500 μM) was prepared by dissolving DIM powder into anhydrous ethanol slowly, stirring until complete dissolution. Complex samples were made by dropping DIM solution slowly into β-LG solution at appropriate levels, resulting DIM concentration ranging from 5 to 20 μM. Ethanol added with DIM never exceed 3% (*v*/*v*) of the total volume. After DIM addition, mixtures were stirred at 275 rpm for 1 h. Stock solutions used for measurements of particle size and zeta potential were prepared to be 50 and 200 μM for β-LG and DIM, respectively. Samples where DIM was present were protected from light throughout the experiments.

### 3.3. Particle Size and Zeta Potential Measurement

Particle size and zeta potential of complex solutions of β-LG (50 μM) and DIM (0–200 μM) were measured by a Zetasizer (Nano-ZS, Malvern Instruments, Malvern, Worcestershire, WR, UK) according to a previous study [42]. Samples were filtrated using a 0.22 μm pore-size filter membrane and ultrasound treated before determination. The parameters were set as follows: solvent viscosity of 0.8872 mPa s, solvent refractive index of 1.33, backscatter of 173° and run time of 10 s for each measurement. Z-average diameter (*D*_h_) and polydispersity index (PDI) were calculated and recorded based on the Stokes‒Einstein equation and zeta potential with the Henry equation.

### 3.4. Absorption Spectra Measurements

The absorption spectra of samples containing protein and various concentration of DIM were recorded in the range of 200–320 nm by a spectrophotometer (UV-2550, Shimadzu, Kyoto, Japan). PB with 20 μM DIM solution was set as control.

### 3.5. Fluorescence Spectra Measurements

Fluorescence spectra of all samples were measured with a spectrofluorometer (RF-5301 PC, Shimatzu Corp., Tokyo, Japan). The emission spectra and synchronous fluorescence emission spectra at wavelength interval of 15 and 60 nm of complex samples were measured in the range of 280 to 440 nm at a constant excitation wavelength (280 nm). PB and PB with 20 μM DIM solutions were set as controls. The binding constant (M^−1^) can be obtained through linear regression from the 1/Δ*F* vs. 1/*C_q_* plot, which corresponds to the following fluorescence enhancing effect Equation [43]:

(1) $$\frac{1}{I_{F}-I^{0}{}_{F}}=\frac{1}{\Delta I_{F\max}}+\frac{1}{K[C_{q}]}\times \frac{1}{\Delta I_{F\max}}$$

where *I*^0^*_F_* is the total protein concentration; *I_F_* is the system with different concentration of DIM; *C_q_* is the concentration of DIM.

### 3.6. Far-Ultraviolet Circular Dichroism (Far-UV CD) Spectroscopy

β-LG (5 μM) and complexes of β-LG and DIM (10 and 20 μM) solutions were determined for secondary structure by a CD spectropolarimeter (MOS-500, Bio-logic, Seyssinet-Pariset, France). The CD spectra of samples contained in a quartz cuvette (optical path of 0.1 cm) were scanned from 190 to 260 nm at room temperature. Mean residue ellipticity ([θ], deg·cm^2^ dmol^−1^) and secondary structure percentage were calculated according to previous study [44] using DichroWeb [45,46] with the CONTIN method.

### 3.7. Fourier Transform Infrared (FT-IR) Spectroscopy

β-LG (5 μM) and complexes of β-LG and DIM (10 and 20 μM) solutions were pre-frozen at −80 °C overnight and then dried at 4 °C for 24 h at 0.3 Mpa. All samples were scanned for FT-IR spectra from 3000 to 1000 cm^−1^ by a FT-IR Spectrometer (IRPrestigeE-21, Shimadzu, Japan) with DRIFT (diffuse reflectance) mode. KBr was dried in the muffle at 140 °C for at least 2 h, and then 200 mg was weighed and mixed with 2 mg sample.

### 3.8. Transmission Electron Microscopy (TEM) Analysis

β-LG, DIM and complexes of β-LG (5 μM) and DIM (20 μM) solutions were observed for their microstructure. Samples (~10 μL) were dropped on a carbon film supported by Cu grid. Filter paper was used to absorb the excessed sample for 1 min. The sample after staining with uranyl acetate (2%, *w*/*v*) solution for 2 min was dried at ambient temperature and then its microstructure was observed with an H-7650 transmission electron microscope (Hitachi High-Technologies, Tokyo, Japan) operated at a 100-kV acceleration voltage.

### 3.9. Molecular Docking

Automated molecular docking between DIM and β-LG was conducted using Discovery Studio 2016 (Accelrys^®^, San Diego, CA, USA). Structures of DIM (Figure S1) and bovine β-LG (3NPO.pdb, Figure S2) were provided by PubChem [47] and Research Collaboratory for Structural Bioinformatics (RCSB) Protein Data Bank [48], respectively.

### 3.10. Data Analysis

Significant differences (*p* = 0.05) between samples in terms of particle size and zeta potential were analyzed using SPSS 21.0 (SPSS Inc., Chicago, IL, USA). The homogeneous data were compared for means using one-way analysis of variance (ANOVA) and then least squared differences (LSD) were applied for the post hoc test. Figures were produced by Origin 2019 (Origin Lab Corporation, Northampton, NC, USA).

## 4. Conclusions

Interactions between β-lactoglobulin (β-LG) and 3,3’-diindolylmethane (DIM) in the model system are studied using spectroscopic analysis and molecular docking. All the results indicated that DIM may bind to β-LG with a relatively high binding constant. Interaction with DIM caused changes in the physiochemical properties and structure of β-LG. The main driving forces for the interactions of β-LG and DIM without heating are hydrophobic and hydrogen bonding. Data from this study may provide useful information for development of whey protein-based DIM nanoparticles for functional food and beverage applications.

## Figures and Tables

**Table 1:** Effects of DIM on Z-average diameter (nm) and zeta potential (mV) of β-LG in solution.

	PDI	*D*_h_	Zeta Potential
50 μM β-LG	0.27 ± 0.01 ^a^	4.86 ± 0.26 ^a^	−8.63 ± 0.50 ^a^
50 μM β-LG + 50 μM DIM	0.32 ± 0.01 ^b^	5.31 ± 0.56 ^ab^	−17.93 ± 0.70 ^b^
50 μM β-LG + 100 μM DIM	0.32 ± 0.01 ^b^	5.82 ± 0.39 ^b^	−17.13 ± 0.87 ^b^
50 μM β-LG + 150 μM DIM	0.33 ± 0.05 ^b^	5.81 ± 0.77 ^b^	−18.03 ± 0.85 ^b^
50 μM β-LG + 200 μM DIM	0.34 ± 0.02 ^b^	5.72 ± 0.31 ^b^	−17.60 ± 0.56 ^b^
PB + 200 μM DIM	-	-	−10.63 ± 0.46 ^c^

Note: DIM is for 3,3′-diindolylmethane; PDI is for polydispersity index. Different lowercase letters denote significant difference between samples with different level of DIM at *p* < 0.05. All samples were determined for triplicate for three batches.

**Table 2:** Effects of DIM on secondary structure (%) of β-LG.

	Helix	Sheet	Turn	Unordered
β-LG	16.15	34.85	22.55	26.50
β-LG/DIM (10 μM)	8.75	38.3	19.35	33.40
β-LG/DIM (20 μM)	5.35	47.9	16.95	29.75

## Supplementary Materials

The following are available online, Figure S1: Structure of 3,3′-diindolylmethane (DIM), Figure S2: Monomeric unit of bovine β-lactoglobulin.
