# Supplementary material for: Interactions between β-Lactoglobulin and 3,3′-Diindolylmethane in Model System
Source: Molecules. 2019 Jun 7;24(11):2151. doi: 10.3390/molecules24112151 (PMC6600512; doi:10.3390/molecules24112151)
Supplement: Supplementary file 1 [file molecules-24-02151-s001.pdf]

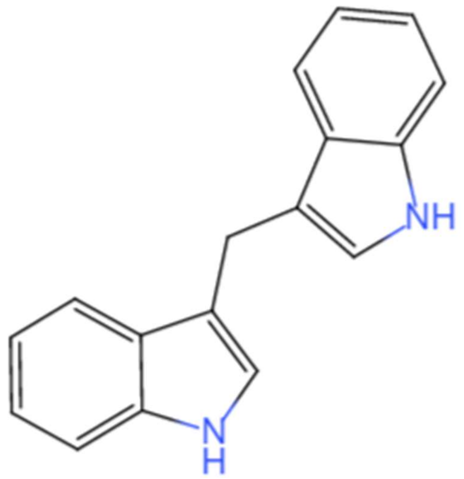

Figure S1. Structure of 3,3'-diindolylmethane (DIM)
Source: PubChem
URL: https://pubchem.ncbi.nlm.nih.gov

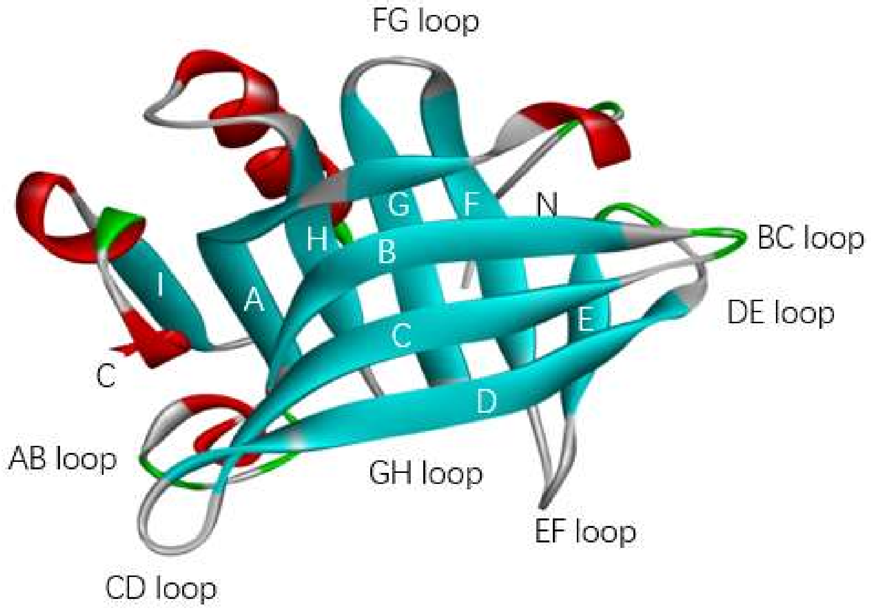

Figure S2. Monomeric unit of bovine β-lactoglobulin. The structure (3uew) was obtained from the Research Collaboratory for Structural Bioinformatics (RCSB) Protein Data Bank (http://www.rcsb.org)
